# Does Point-of-care Ultrasound Use Impact Resuscitation Length, Rates of Intervention, and Clinical Outcomes During Cardiac Arrest? A Study from the Sonography in Hypotension and Cardiac Arrest in the Emergency Department (SHoC-ED) Investigators

**DOI:** 10.7759/cureus.4456

**Published:** 2019-04-13

**Authors:** Paul R Atkinson, Nicole Beckett, James French, Ankona Banerjee, Jacqueline Fraser, David Lewis

**Affiliations:** 1 Emergency Medicine, Saint John Regional Hospital/Dalhousie University, Saint John, CAN; 2 Internal Medicine, Saint John Regional Hospital/Dalhousie University, Saint John, CAN; 3 Miscellaneous, WorkSafeNB, Saint John, CAN

**Keywords:** point of care ultrasound, acls, cardiac arrest, shoced

## Abstract

Introduction

This third study in the Sonography in Hypotension and Cardiac Arrest in the Emergency Department (SHoC-ED) series examined potential relationships between point-of-care ultrasound (PoCUS) use and the length of resuscitation, the frequency of interventions, and clinical outcomes during cardiac arrest.

Methods

A health records review was completed for adult patients (>19 years, without a do not resuscitate (DNR) order) who presented to a tertiary emergency department in cardiac arrest between 2010 and 2014. Patients were grouped based on PoCUS use and findings for cardiac activity. Data were analyzed for length of resuscitation, frequency of interventions, return of spontaneous circulation (ROSC), survival to hospital admission (SHA), and survival to hospital discharge (SHD).

Results

Of the 223 patients who met inclusion criteria, 180 (80.7%) received assessment by PoCUS during cardiac arrest management in the emergency department (ED). In the PoCUS group, 21 (11.6%) demonstrated cardiac activity and 159 (88.4%) did not. Patients with activity on PoCUS had longer mean resuscitation times (27.3; 95% confidence interval 17.7-37.0 min) than patients with no activity (11.51; 10.2-12.8 min) and patients who did not receive a PoCUS exam (14.36; 9.89-18.8 min). Patients with cardiac activity on PoCUS were more likely to receive endotracheal intubation (ET; 95.23%; 86.13-104.35%) and epinephrine (Epi; 100%; 100-100%) than patients with no activity (ET: 46.54%; 38.8-54.3%; Epi: 82.39%; 76.50-88.31%) and those with no PoCUS (ET: 65.11%; 50.87-79.36%; Epi: 81.39%; 69.76-93.03%). Those with no cardiac activity on PoCUS were much less likely to achieve ROSC (19.5%; 13.4-25.6), SHA (6.9%; 2.97-10.86%) and SHD (0.6%; -0.5-1.8%) compared to those with cardiac activity on PoCUS (ROSC; 76.19%; 57.97-94.4%), SHA (33.3%; 13.2-53.5%), SHD (9.5%; -3-22.07%), and those with no PoCUS (ROSC 39.5%; 24.9-54.1%; SHA 27.9%; 14.5- 41.3%, and SHD 6.9%; -0.6-14.59).

Conclusions

Emergency department cardiac arrest patients with cardiac activity on PoCUS received longer resuscitation with higher rates of intervention as compared to those with negative findings or when no PoCUS was performed. Patients with cardiac activity on PoCUS had improved clinical outcomes as compared with patients not receiving PoCUS, and patients with no activity on PoCUS.

## Introduction

Although advanced cardiac life support (ACLS) algorithms do not currently mandate the use of echocardiography [1], cardiac point-of-care ultrasound (PoCUS) is now recognized in international resuscitation guidelines and is widely used during cardiac arrest management in emergency departments and critical care settings [2-7]. Physicians use PoCUS to identify not only findings such as pericardial effusion, hypovolemia, cardiac tamponade, and pulmonary embolus as potential causes of the arrest but also to confirm the presence or absence of cardiac activity [8]. In some cases, these findings may lead to changes in therapeutic management such as pericardiocentesis, thrombolysis, or rapid volume infusion [9].

Previous studies suggest that in addition to predicting outcome in cardiac arrest, PoCUS may be useful in identifying patients who may respond to more aggressive resuscitation efforts, as well as in aiding the decision to terminate resuscitation [10-13].

In this study, the third in the Sonography in Hypotension and Cardiac Arrest in the Emergency Department (SHoC-ED) series, we wished to compare the resuscitative effort, including the length of resuscitation and frequency of interventions, in addition to the clinical outcomes of return of spontaneous circulation (ROSC) and survival to hospital discharge (SHD) in patients receiving standard ACLS management with and without cardiac PoCUS. In addition, we wished to examine the relationship between visualizing cardiac activity on PoCUS and these outcomes. We plan to report the diagnostic validity of PoCUS in cardiac arrest separately.

## Materials and methods

Study settings

A health records review was completed for patients who presented in cardiac arrest to the Saint John Regional Hospital (SJRH) emergency department (ED), a tertiary healthcare center in New Brunswick, Canada, between 2010 and 2014.

Subject selection

All adult cardiac arrest patients brought to the emergency department during the study period were considered for inclusion. Patients were excluded if they were under the age of 19 years, resuscitation was halted due to end-of-life decisions, or for the initiation of cardiac arrest as an inpatient. Patients were grouped based on whether they received a PoCUS assessment during ACLS or not. The PoCUS group was further sub-divided based on visualized cardiac activity or cardiac standstill on the initial PoCUS examination. A waiver of consent was granted, as many of the study participants were deceased and it would be non-empathetic to contact families for consent. This project was approved by the Research Ethics Board for the Horizon Health Network (File No. 2015-2132).

Protocol for resuscitation

Resuscitation, delivered as routine care, was guided by ACLS protocols and institutional policies. PoCUS was performed during designated pauses, such as pulse and rhythm checks and necessary resuscitative procedures (e.g. intubation), so as to minimize cardiopulmonary resuscitation (CPR) interruption. Pauses were minimized as per ACLS recommendations, however, actual delays in CPR were not recorded.

Images were acquired using the standard PoCUS technique, using curvilinear or phased array ultrasound probes. Ultrasound views included sub-xiphoid, parasternal long axis, or apical four chambers. Image requirements were based on adequate echocardiographic windows and image quality, as determined by the physician performing the bedside ultrasound. For patients that were difficult to image, a combination of views was used to obtain adequate information. Sonographic images were obtained by competent personnel with experience in PoCUS; findings were communicated to the team leader.

Cardiac activity on PoCUS was defined as sustained coordinated contractility of the left ventricle, with visible valve movement.

Study outcomes

The primary outcome measure was a resuscitative effort as evidenced by length-of-resuscitation and frequency-of-interventions such as rates of administration of epinephrine and endotracheal intubation. Secondary clinical outcomes were rates of return-of-spontaneous-circulation (ROSC) and survival-to-hospital-discharge (SHD).

Data collection

The data for this study were obtained through a structured chart review in line with the REporting of studies Conducted using Observational Routinely-collected health Data (RECORD) statement guidelines [14]. The ED cardiac arrest database was used to identify all cardiac arrest patients. In addition, full patient charts (ambulance charts, emergency department charts, cardiac arrest records, electronic records, and inpatient charts) for patients with a presentation of cardiac arrest were analyzed. Subject data, with protected health information (PHI) removed, were stored in a local database. The local site kept secured records to enable the identification of the patient source if a data review was required.

Patient information included the following: past medical history, events surrounding the cardiac arrest, actions taken by health care professionals, peri-arrest presentation, peri-arrest interventions, and patient outcomes. Recorded health care professional actions included ACLS medication administration, airway management, chest compressions, defibrillation, pacing, and other resuscitative interventions.

Statistical analysis

Study sample size was determined by clinical outcome measures, with a minimum sample size of 185 required to detect a small difference (5%) from a baseline population survival to hospital discharge rate of 5%, with a power of 0.8 and an alpha of 0.05. Point estimates and proportions are reported with appropriate confidence intervals. Categorical data were analyzed using Fisher’s Exact Test and continuous data with Kruskal Wallis non-parametric test. The software used for data analysis was R (R Core Team (2017). R: A language and environment for statistical computing. R Foundation for Statistical Computing, Vienna, Austria).

## Results

Baseline characteristics

Of the 223 patients who met inclusion criteria, 180 (80.7%) received an assessment by PoCUS during cardiac arrest management in the emergency department (ED). In the PoCUS group, 21 (11.6%) demonstrated cardiac activity and 159 (88.4%) did not. Baseline characteristics were similar for each group and are shown in Table 1.

**Table 1:** Baseline population characteristics Kruskal-Wallis non-parametric test, two-tailed; Fisher’s Exact Test, two-tailed PoCUS: point-of-care ultrasound; N: number; SD: standard deviation. CI: confidence interval; CPR: cardiopulmonary resuscitation

Variable		No PoCUS	Positive Cardiac Activity on PoCUS	Negative Cardiac Activity on PoCUS	P-value
Age	Mean years +/- SD (N)	65.67±5.58	65.81±3.52	65.19±3.86	0.98
Sex	Male n/N (%; 95% CI)	29/43 (67.4; 53.43-81.45%)	9/21 (42.86; 21.69-64.03%)	111/159 (69.81; 62.67-76.95%)	0.05*
Witnessed arrest	n/N (%; 95% CI)	30/42 (71.43; 57.77-85.09%)	16/21 (76.19; 57.97-94.41%)	100/158 (63.29; 55.77-70.81%)	0.42
Bystander CPR	n/N (%; 95% CI)	26/41 (63.41; 48.67-78.15%)	15/18 (83.33; 66.12-100.5%)	102/152 (67.10; 59.64-74.57%)	0.32
Arrival by ambulance	n/N (%) [95% CI]	42/43 (97.67) [93.17-102.18]	21/21 (100) [100-100]	157/159 (98.74) [97.01-100.47]	0.64

Resuscitation effort

Patients with cardiac activity on PoCUS received a longer mean duration of resuscitation than those with no cardiac activity (27.33; 95% confidence interval 17.7-37.0 min vs. 11.51; 10.2-12.8 min) than patients who did not receive PoCUS (14.36; 9.89-18.8 min; p=0.001). A similar pattern was seen for interventions, with a higher rate of endotracheal intubation in patients with cardiac activity on PoCUS compared to those with no cardiac activity on PoCUS (95.23%; 86.13-104.35 vs. 46.54%; 38.79-54.29) and those with no PoCUS (65.11%; 50.87-79.36; p<0.001). A greater proportion of patients with cardiac activity on PoCUS received epinephrine as compared to those with no cardiac activity on PoCUS (100%; 100-100 vs. 82.39%; 76.5-88.3) and those who did not receive PoCUS (81.39%; 69.76-93.03; <0.001). Table 2 shows outcomes for resuscitative effort, including the length of resuscitation and the number of interventions.

**Table 2:** Resuscitation effort and interventions ** Kruskal-Wallis test, p<0.05; * Fisher’s Exact Test, two-tailed, p<0.05 PoCUS: point-of-care ultrasound; N: number; CI: confidence interval

Outcome		No PoCUS	Positive Cardiac Activity on PoCUS	Negative Cardiac Activity on PoCUS	P-value
Resuscitation time	Minutes (95% CI)	14.36 (9.89-18.8)	27.33 (17.7-37.0)	11.51 (10.2-12.8)	0.001**
Endotracheal intubation	n/N (%; 95% CI)	28/43 (65.11; 50.87-79.36%)	20/21 (95.23; 86.13-104.35%)	74/159 (46.54; 38.79-54.29%)	<0.001*
Epinephrine administration	n/N (%; 95% CI)	35/43 (81.39; 69.76-93.03%)	21/21 (100; 100-100%)	131/159 (82.39; 76.50-88.31%)	<0.001*

Clinical outcomes

Patients who received a PoCUS exam demonstrating cardiac activity had significantly higher rates of ROSC (76.19%; 57.97-94.4%) than patients with no activity on PoCUS (19.5%; 13.4-25.6%) and than patients who did not undergo PoCUS examination (39.5%; 24.9-54.1; p<0.001). Rates of survival to hospital admission were higher in patients with cardiac activity on PoCUS (33.3%; 13.2-53.5) than in those with no activity (6.9%; 2.97-10.86%; p<0.001). Patients with cardiac activity on PoCUS also had higher rates of survival to hospital discharge (9.5%; -3-22.07%) than those with no activity (0.6%; -0.5-1.8%; p=0.008). There was no survival to hospital admission or discharge advantage over patients who did not undergo a PoCUS examination. Further details on clinical outcomes are shown in Table 3.

**Table 3:** Clinical outcomes * Fisher’s exact test, two-tailed, p<0.05. PoCUS: point-of-care ultrasound; N: number; CI: confidence interval; ROSC: return of spontaneous circulation; SHA: survival to hospital admission; SHD: survival to hospital discharge

Outcome		No PoCUS	Positive Cardiac Activity on PoCUS	Negative Cardiac Activity on PoCUS	P-value
ROSC	n/N (%; 95% CI)	17/43 (39.5; 24.9-54.1%)	16/21 (76.19; 57.97-94.4%)	31/159 (19.5; 13.4-25.6%)	<0.001*
SHA	n/N (%; 95% CI)	12/43 (27.9; 14.5-41.3)	7/21 (33.3; 13.2-53.5%)	11/159 (6.9; 2.97-10.86%)	<0.001*
SHD	n/N (%; 95% CI)	3/43 (6.9; -0.6-14.59%)	2/21 (9.5; -3-22.07%)	1/159 (0.6; -0.5-1.8%)	0.008*

## Discussion

This study compared resuscitation effort, including the length of resuscitation and number of interventions, as well as clinical outcomes in patients who did and did not receive cardiac PoCUS during emergency department cardiac arrest management.

Our findings indicate increased length of resuscitation and frequency of epinephrine and intubation use in patients with positive cardiac activity on PoCUS when compared to both patients without cardiac activity and patients not receiving PoCUS evaluation. This suggests that emergency physicians and the resuscitation team provide increased effort (perhaps unknowingly) for patients when cardiac activity is seen on PoCUS and stop resuscitation earlier for patients when no PoCUS was performed or when there was no evidence of ongoing cardiac activity. This behavior is consistent with recommendations from previous studies that have suggested that an absence of cardiac activity on PoCUS can aid in the decision-making for resuscitation termination [11-13], however, this advice is not consistent with current resuscitation guidelines [1]. It is also consistent with previous reports that activity seen on PoCUS may be associated with improved outcomes from increased use of inotropes [13].

The findings of this study also suggest that in addition to receiving additional resuscitative effort, there may be improvements in rates of ROSC, survival to hospital admission, and survival to hospital discharge in patients with cardiac activity on PoCUS. This advantage in short-term and longer-term survival was seen in patients undergoing ACLS with an initial PoCUS examination where cardiac activity was visualized as compared with those who had cardiac standstill. The survival advantage was seen only for ROSC when comparing patients with cardiac activity on PoCUS to patients who did not undergo a PoCUS exam. In addition, rates of survival to hospital discharge were actually higher in both the groups, with cardiac activity on and the group with no PoCUS compared to the group of patients with cardiac standstill on PoCUS.

Our findings are consistent with previous studies that have shown increased rates of survival in patients with cardiac activity on PoCUS [12]. They also suggest that seeing cardiac activity on PoCUS results in prolonged resuscitation efforts, with a higher rate of interventions such as intubation and administration of epinephrine. It is difficult to know if the improved outcomes recorded are due to the increased resuscitative effort or if PoCUS simply identifies those patients who are more likely to survive. The short-term survival benefits for patients who have cardiac activity seen on PoCUS over those not receiving PoCUS, in addition to the long-term survival benefits over those with cardiac standstill on PoCUS suggest that the use of PoCUS during cardiac arrest may have a direct impact on clinical outcomes.

Physicians seem to provide similar levels of effort in patients not receiving PoCUS and those with cardiac standstill on PoCUS while providing increased effort and seeing improved outcomes in patients demonstrating initial cardiac activity on PoCUS. However, it is important to emphasize that completion of PoCUS must occur within the 10-second pulse check window, avoiding unnecessary delays in chest compressions that could be associated with worse outcomes [15].

Limitations include the retrospective observational non-randomized nature of our comparison, the relatively low survival rate, and a lack of control for the quality of PoCUS performed. As such, we believe that this work provides the basis for moving toward a randomized controlled trial of PoCUS in cardiac arrest.

## Conclusions

Patients with cardiac activity visualized on PoCUS had longer resuscitation attempts, more frequent interventions, as well as higher rates of ROSC than patients with cardiac standstill on PoCUS and patients not receiving a PoCUS exam. Long-term survival rates were higher in patients with cardiac activity on PoCUS than in those demonstrating cardiac standstill. A randomized controlled trial of PoCUS in cardiac arrest may be indicated.

## Appendices

Clinicians’ capsule

What Is Known About the Topic?

Point-of-care ultrasound (PoCUS) is used during cardiac arrest in the emergency department to detect cardiac activity.

What Did This Study Ask?

What is the relationship between PoCUS use and length of resuscitation, frequency of interventions, and clinical outcomes during cardiac arrest?

What Did This Study Find?

Patients with cardiac activity on PoCUS received increased resuscitative effort and had improved clinical outcomes as compared to those with negative findings or when no PoCUS was performed.

Why Does This Study Matter to Clinicians?

The use of PoCUS during resuscitation may help to identify patients who may benefit from an increased resuscitative effort.

## References

[1] Olasveengen TM, de Caen AR, Mancini ME (2017). 2017 international consensus on cardiopulmonary resuscitation and emergency cardiovascular care science with treatment recommendations summary. Resuscitation.

[2] American College of Emergency Physicians (2009). Emergency ultrasound guidelines. Ann Emerg Med.

[3] (2019). Royal College of Emergency Medicine. Emergency medicine ultrasound level 1 training. https://www.rcem.ac.uk/RCEM/Event_Display.aspx?EventKey=CUS181008&WebsiteKey=b3d6bb2a-abba-44ed-b758-467776a958cd.

[4] Henneberry RJ, Hanson A, Healey A (2012). Use of point of care sonography by emergency physicians. CAEP Ultrasound Position Statement Working Group. Can J Emerg Med.

[5] Atkinson P, Bowra J, Milne J (2017). International Federation for Emergency Medicine Consensus Statement. Sonography in hypotension and cardiac arrest (SHoC). An international consensus on the use of point of care ultrasound for undifferentiated hypotension and during cardiac arrest. CJEM.

[6] (2019). Focused echocardiography in emergency life support (FEEL-UK). https://www.resus.org.uk/information-on-courses/focused-echocardiography-in-emergency-life-support/.

[7] Hayhurst C, Lebus C, Atkinson PR (2011). An evaluation of echo in life support (ELS); is it feasible? What does it add?. Emerg Med J.

[8] Hernandez C, Shuler K, Hannan H, Sonyika C, Likourezos A, Marshall J (2008). C.A.U.S.E.: cardiac arrest ultra-sound exam - a better approach to managing patients in primary non-arrhythmogenic cardiac arrest. Resuscitation.

[9] Andrus P, Dean A (2013). Focused cardiac ultrasound. Global Heart.

[10] Blyth L, Atkinson P, Gadd K, Lang E (2012). Bedside focused echocardiography as predictor of survival in cardiac arrest patients; a systematic review. Acad Emerg Med.

[11] Tsou PY, Kurbedin J, Chen YS (2017). Accuracy of point-of-care focused echocardiography in predicting outcome of resuscitation in cardiac arrest patients: a systematic review and meta-analysis. Resuscitation.

[12] Gaspari R, Weekes A, Adhikari S (2016). Emergency department point-of-care ultrasound in out-of-hospital and in-ED cardiac arrest. Resuscitation.

[13] Gaspari R, Weekes A, Adhikari S (2017). A retrospective study of pulseless electrical activity, bedside ultrasound identifies interventions during resuscitation associated with improved survival to hospital admission. A REASON study. Resuscitation.

[14] Benchimol EI, Smeeth L, Guttmann A (2015). The reporting of studies conducted using observational routinely-collected health data (RECORD) statement. PLoS Med.

[15] in't Veld MA, Allison MG, Bostick DS, Fisher KR, Goloubeva OG, Witting MD, Winters ME (2017). Ultrasound use during cardiopulmonary resuscitation is associated with delays in chest compressions. Resuscitation.

